# The Antihypertensive Guanabenz Exacerbates Integrated Stress Response and Disrupts the Brain Circadian Clock

**DOI:** 10.3390/clockssleep5040043

**Published:** 2023-10-31

**Authors:** Hao Lin, Muhammad Naveed, Aidan Hansen, Tracy G. Anthony, Ruifeng Cao

**Affiliations:** 1 Department of Neuroscience and Cell Biology, Robert Wood Johnson Medical School, Rutgers University, Piscataway, NJ 08854, USA; hao.lin2022@rutgers.edu; 2 Department of Biomedical Sciences, University of Minnesota Medical School, Duluth, MN 55812, USA; drnoveed@aol.com (M.N.); hans7656@umn.edu (A.H.); 3 Department of Nutritional Sciences, The New Jersey Institute for Food, Nutrition, and Health, Rutgers University, Piscataway, NJ 08854, USA; tracy.anthony@rutgers.edu; 4 Department of Neurology, Robert Wood Johnson Medical School, Rutgers University, Piscataway, NJ 08854, USA

**Keywords:** circadian rhythm, guanabenz, integrated stress response, eIF2α, SCN

## Abstract

The circadian clock regulates a variety of biological processes that are normally synchronized with the solar day. Disruption of circadian rhythms is associated with health problems. Understanding the signaling mechanisms that couple cell physiology and metabolism to circadian timekeeping will help to develop novel therapeutic strategies. The integrated stress response (ISR) is activated by the cellular stressors to maintain physiological homeostasis by orchestrating mRNA translation. Aberrant ISR has been found in a number of neurological diseases that exhibit disrupted circadian rhythms and sleep. Recent work has started to uncover a critical role for the ISR in regulating the physiology of the circadian clock. Guanabenz (2,6-dichlorobenzylidene aminoguanidine acetate) is an orally bioavailable α2-adrenergic receptor agonist that has been used as an antihypertensive for decades. Recent studies demonstrated that guanabenz can regulate the ISR. Here, we assessed the effects of guanabenz on cellular and behavioral circadian rhythms using a multidisciplinary approach. We found that guanabenz can induce the ISR by increasing eIF2α phosphorylation in cultured fibroblasts as well as in the mouse brain. The hyperphosphorylation of eIF2α by guanabenz is associated with the shortened circadian period in cells and animals and the disruption of behavioral circadian rhythms in mice. Guanabenz administration disrupted circadian oscillations of the clock protein Per1 and Per2 in the mouse suprachiasmatic nucleus, the master pacemaker. These results uncover a significant yet previously unidentified role of guanabenz in regulating circadian rhythms and indicate that exacerbated ISR activation can impair the functions of the brain’s circadian clock by disrupting clock gene expression.

## 1. Introduction

The biological clock regulates the sleep–wake cycle as well as many other biological processes in a variety of living organisms including humans [1]. As a result, numerous physiological and metabolic processes exhibit approximately 24 h (circadian) rhythmicity that is normally synchronized with the solar day [2]. Disruption of circadian rhythms can cause health problems including jet lag and shift-work sleep disorders [3]. More importantly, circadian clock dysfunctions are associated with common human diseases, including neurodegenerative disorders, metabolic syndromes, cardiovascular disease, and cancer [4]. Thus, understanding biological clock mechanisms will help to develop novel therapeutic strategies and identify new molecular targets to treat these diseases. 

The circadian system is hierarchically organized. In contrast to the mammalian master pacemaker, which is located in the hypothalamic suprachiasmatic nucleus [5], clock genes are expressed in almost all body cells [6,7]. The cellular clock is intrinsically driven by transcriptional/translational feedback loops (TTFL, [2,8]). In a negative feedback loop, the mammalian clock proteins Bmal1 and Clock form complexes and activate transcription of the *Period* (*Per1*, *2*, and *3*) and *Cryptochrome* (*Cry1* and *2*) via the E-boxes. The Per/Cry protein complexes accumulate in the cytosol and then translocate to the nucleus to suppress their own gene expression [9]. Beyond the TTFL, emerging evidence supports significant crosstalk between the molecular clock machinery and the intracellular signaling network. The crosstalk mechanisms that couple cell physiology and metabolism to circadian timekeeping are complex and remain to be fully understood [10]. 

The integrated stress response (ISR) is a conserved intracellular signaling network that enables cells to adapt to variable environments in order to maintain health [11,12,13]. ISR is activated by a variety of cellular stressors and functions to maintain physiological homeostasis by tuning down general mRNA translation while augmenting the synthesis of specific proteins that drive a new transcriptional program. The cellular stressors are sensed by ISR regulators, which are specialized protein kinases, including general control non-derepressible 2 (GCN2), protein kinase R-like endoplasmic reticulum kinase (PERK), protein kinase RNA-activated (PKR) and heme-regulated inhibitor (HRI). Their kinase activities converge at the level of phosphorylation of eukaryotic translation initiation factor 2 alpha (eIF2α) to alter mRNA translation and reprogram gene expression [14,15]. The ISR regulates important neural processes such as stem cell differentiation, synaptic plasticity, learning and memory, and food uptake [12]. Recent work has started to uncover the critical roles of the ISR in regulating the circadian clock physiology in multiple organisms [16,17]. The ISR can be overactivated under pathological conditions, but the effects of ISR hyperactivation on the circadian clock remain unknown.

Guanabenz (2,6-dichlorobenzylidene aminoguanidine) is an orally bioavailable α2-adrenergic receptor agonist that is approved to treat hypertension [18]. Recent in vitro studies demonstrate that guanabenz may enhance the ISR by inhibiting the binding of the eIF2 phosphatase GADD34 to PP1, thereby inhibiting the dephosphorylation of p-eIF2α [19]. Guanabenz readily crosses the blood–brain barrier [20]. However, its effects on the brain’s circadian system have never been studied. In the current study, we assessed the effects of guanabenz on cellular and behavioral circadian rhythms using a multidisciplinary approach. We found that guanabenz increased eIF2α phosphorylation in cultured cells as well as in the mouse brain, including the SCN. The hyperphosphorylation of eIF2α is associated with shortened circadian periods in cells and disrupted behavioral rhythms in mice. These results demonstrate a significant, albeit previously unidentified, role of guanabenz in circadian regulation and indicate that exacerbated ISR disrupts the circadian clock function in cells and animals. 

## 2. Results

### 2.1. Guanabenz Increases eIF2α Phosphorylation in 3T3 Fibroblasts

**Figure 1:**
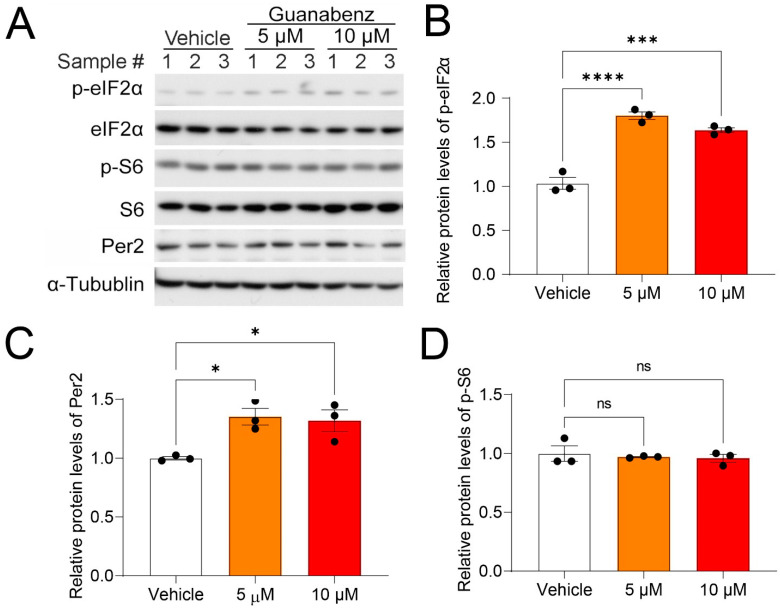
Guanabenz treatment increases eIF2α phosphorylation in the 3T3 fibroblasts: (**A**) Western blots from 3T3 fibroblast lysates. For this experiment, 3T3 fibroblasts were treated with 5 µM or 10 µM of guanabenz for 8 h. Note that guanabenz treatment increased phospho-eIF2α levels but not phospho-S6 levels in the fibroblasts. Per2 levels were also moderately increased by the guanabenz treatment. α-Tubulin was used as a loading control. See Figure S1 for full-length western blotting images. Quantitative analysis of the blot intensities is shown in (**B**) for phospho-eIF2α (F_(2,6)_ = 68.23, *p* < 0.0001, one-way ANOVA; Vehicle vs. 5 μM, t = 11.10, *p* < 0.0001; Vehicle vs. 10 μM, t = 8.708, *p* = 0.0004; Bonferroni’s post hoc comparisons), in (**C**) for Per2 (F_(2,6)_ = 8.256, *p* = 0.0189, one-way ANOVA; Vehicle vs. 5 μM, t = 3.684, *p* = 0.0308; Vehicle vs. 10 μM, t = 3.327, *p* = 0.0476; Bonferroni’s post hoc comparisons), and in (**D**) for *p*-S6 (F_(2,6)_ = 0.2358, *p* = 0.7968, one-way ANOVA; Vehicle vs. 5 μM, t = 0.0475, *p* > 0.9999; Vehicle vs. 10 μM, t = 0.6671, *p* > 0.9999; Bonferroni’s post hoc comparisons). (**B**–**D**) The data are shown as individual values and mean ± SEM. * *p* < 0.05 *** *p* < 0.001, **** *p* < 0.0001; ns, not significant.

To substantiate earlier work demonstrating the ISR-enhancing effects of guanabenz, we first assessed the effects of guanabenz on the activities of translational control pathways in cultured 3T3 fibroblasts. Using Western blotting, we found that treatment with guanabenz (5 µM and 10 µM) for 8 h significantly increased the relative levels of phosphorylated eIF2α (p-eIF2α) over the total eIF2α, indicating increased eIF2α phosphorylation (Figure 1A,B). The levels of the clock protein Per2 were also moderately increased (Figure 1A,C). In contrast, the relative levels of phosphorylated S6 (p-S6), an indicator of the mechanistic target of the rapamycin complex 1 signaling (mTORC1) pathway, over the total S6 were not changed (Figure 1A,D), indicating significant effects of guanabenz on the eIF2 but not on the mTORC1 pathway. As a loading control, the levels of α-tubulin were not changed. Together, these results demonstrate that guanabenz activates the ISR in fibroblasts without altering other translational control pathways such as mTORC1. 

### 2.2. Guanabenz Shortens the Circadian Period Length and Increases the Amplitude of Per2-dLuc Rhythms in 3T3 Fibroblasts

**Figure 2:**
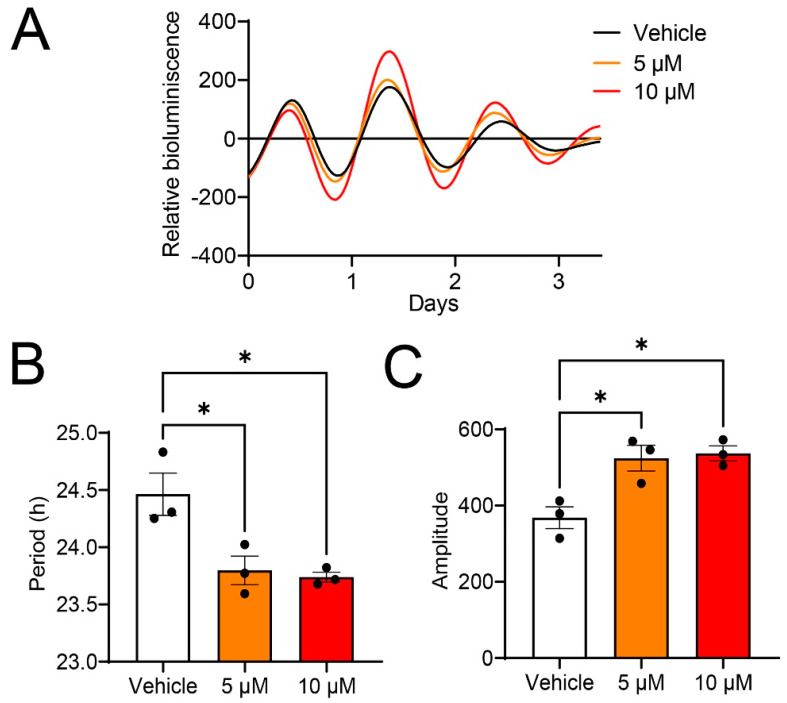
Guanabenz shortens the circadian period and increases the amplitude of Per2-dLuc rhythms in 3T3 fibroblasts: (**A**) Representative traces of bioluminescence recordings. For this experiment, 3T3 *Per2-dLuc* fibroblasts were treated with 5 µM or 10 µM guanabenz, or the vehicle (DMSO), and bioluminescence recordings were performed using the LumiCycle instrument. (**B**) Circadian period of *Per2-dLuc* rhythms. Note that guanabenz treatment shortened the circadian period (F_(2,6)_ = 9.451, *p* = 0.0140, one-way ANOVA; Vehicle vs. 5 μM, t = 3.597, *p* = 0.0342; Vehicle vs. 10 μM, t = 3.913, *p* = 0.0236; Bonferroni’s post hoc comparisons). (**C**) Amplitude of Per2-dLuc rhythms. Note that guanabenz treatment increased the amplitude (F_(2,6)_ = 11.25, *p* = 0.0093, one-way ANOVA; Vehicle vs. 5 μM, t = 3.942, *p* = 0.0228; Vehicle vs. 10 μM, t = 4.255, *p* = 0.0161; Bonferroni’s post hoc comparisons). Data are shown as individual values and mean ± SEM. * *p* < 0.05.

Next, we determined the effects of guanabenz on the molecular circadian rhythms in 3T3 fibroblasts with a *Per2-dLuc* luciferase reporter, which are widely used to study the peripheral clock functions. Using bioluminescence recording, we recorded the *Per2-dLuc* rhythms in 3T3 cells. Interestingly, we found that the guanabenz treatment (5 µM and 10 µM) led to a significant decrease in the circadian period as compared to the vehicle (DMSO) treatment (Figure 2A,B). Guanabenz treatment also moderately but significantly increased the amplitude of *Per2-dLuc* rhythms (Figure 2A,C). Together, these results demonstrate the significant effects of guanabenz on cellular circadian rhythms. The effects are consistent with the established role of eIF2α phosphorylation in promoting *Per2* expression [16].

### 2.3. Oral Administration of Guanabenz Increases eIF2α Phosphorylation in the Mouse Brain

**Figure 3:**
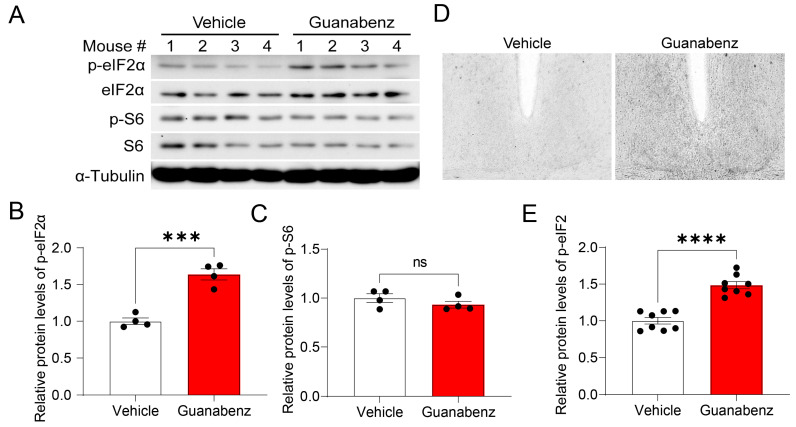
Guanabenz administration increases eIF2α phosphorylation in the mouse brain: (**A**) Western blots from mice brain lysates. For this experiment, mice were given guanabenz (11 mg/mL) in drinking water for 7 d, and brain tissue was used for Western blotting. Note that guanabenz treatment increased the levels of phospho-eIF2α proteins but not phospho-S6 proteins. α-Tubulin was used as a loading control. See Figure S1 for full-length western blotting images. The quantitative analysis of the blots is shown in (**B**) for p-eIF2α and (**C**) for p-S6. Note that eIF2α phosphorylation (t_(6)_ = 7.321, *p* = 0.0003, Student’s *t*-test), but not the S6 phosphorylation (t_(6)_ = 1.205, *p* = 0.6998, Student’s *t*-test), was increased in the guanabenz-treated group. (**D**) Representative microscopic images of immunostaining for p-eIF2α in the SCN. The quantitative analysis of the immunostaining intensity for p-eIF2α is shown in (**E**). Note that guanabenz treatment significantly increased the levels of p-eIF2α in the SCN (t_(14)_ = 7.295, *p* < 0.0001, Student’s *t*-test). Data are shown as individual values and mean ± SEM. *** *p* < 0.001, **** *p* < 0.0001; ns, not significant.

To study the in vivo effects of guanabenz, C57BL/6J mice were administered guanabenz in drinking water (11 mg/mL) for 7 d and then sacrificed. Brain tissue was examined for eIF2α and S6 phosphorylation and expression using Western blotting. Strikingly, we found that phosphorylated eIF2α, but not the total eIF2α, was significantly increased in the mouse brain treated with guanabenz as compared to the control group treated with regular water, indicating increased eIF2α phosphorylation (Figure 3A,B). In contrast, the levels of phosphorylated S6 (p-S6) or total S6 were not changed by guanabenz treatment (Figure 3A,C). Using immunostaining, we deleted the level of phosphorylated eIF2α in the SCN and found a significant increase in phosphorylated eIF2α in the SCN of mice treated with guanabenz as compared to the control (Figure 3D,E). These results are consistent with the effects of guanabenz in 3T3 cells (Figure 1) and demonstrate that guanabenz can trigger the ISR in multiple tissues.

### 2.4. Oral Administration of Guanabenz Shortens the Circadian Period and Disrupts Behavioral Rhythms in Mice

**Figure 4:**
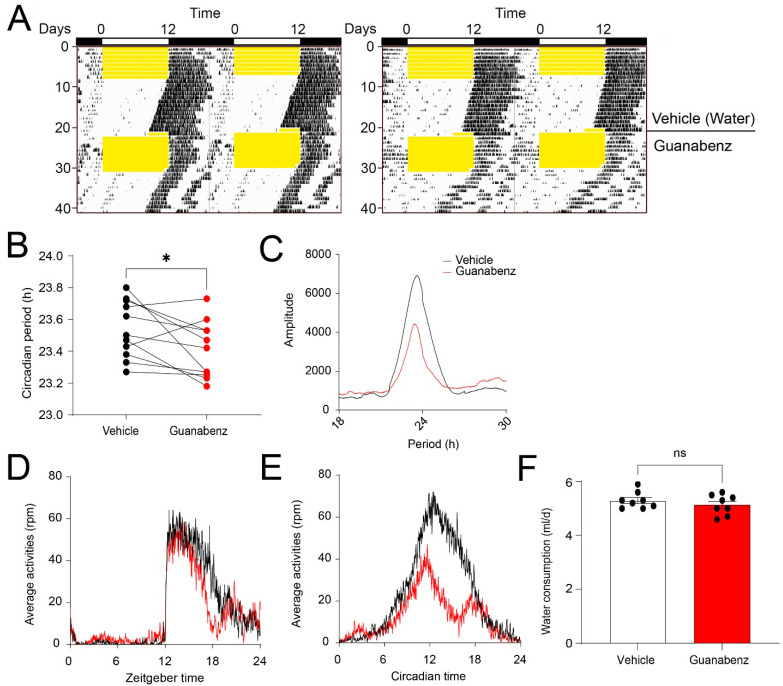
Guanabenz administration shortens the circadian period and disrupts behavioral rhythms in mice: (**A**) Representative double-plotted actograms of wheel-running activities from C57BL/6J mice. For this experiment, animals given regular drinking water were kept in a 12 h/12 h LD cycle for 7 days and transferred to DD for 14 days. Next, the mice were given guanabenz (11 mg/mL) in the drinking water and kept in a 12 h/12 h LD cycle for 7 days followed by a DD cycle for 14 days. Yellow shades indicate light periods. Note that the free-running period was decreased through guanabenz treatment. (**B**) Circadian period in DD before and after guanabenz treatment (t_(10)_ = 2.337, *p* = 0.0416, paired *t*-test). * *p* < 0.05. (**C**) The pooled periodograms in DD from all mice before and after guanabenz treatment. Note that the circadian rhythmicity was weakened after guanabenz treatment, as indicated by a lower amplitude of the peak of the periodogram. The average amount of wheel-running activities in LD (**D**) and DD (**E**) from all the mice was quantified. Note that guanabenz treatment decreased wheel-running activities in the dark phase both in LD and DD. Eleven mice were used in this experiment. (**F**) A bar graph indicating the daily water consumption of the mice. Note that the water consumption was not significantly different between the vehicle and guanabenz-treated group (t_(14)_ = 0.9499, *p* = 0.3583, unpaired *t*-test). Data are shown as individual values and mean ± SEM. ns, not significant.

Next, to determine whether guanabenz regulates circadian rhythms in mice, we recorded the wheel-running behavior of C57BL/6J mice. Mice given regular drinking water were first kept in a 12 h/12 h light–dark (LD) cycle for 7 d and then transferred to and kept in continuous darkness (DD) for 14 d. Next, the mice were given guanabenz (11 mg/mL) in the drinking water and kept in LD for 7 d and then in DD for 14 d. The mice were entrained to LD and free-ran in DD with regular water, as expected. In the presence of guanabenz water, mice re-entrained to LD and free-ran in DD. Interestingly, however, the circadian period in DD was significantly shortened by guanabenz administration, and the rhythms in DD were disrupted (Figure 4A,B). Instead of showing a consolidated bout of wheel-running activities starting at the onset of circadian night, mice treated with guanabenz exhibited two fragmented bouts of wheel-running activities in the subjective night and showed more wheel-running activities in the subjective day (Figure 4A). As a result, the amplitude of the periodogram in DD was decreased after guanabenz treatment (Figure 4C). The average activities were also decreased in both LD and DD after guanabenz treatment. Activities at night were split into two bouts both in LD and in DD (Figure 4D,E). The amount of water consumption was not changed with the addition of guanabenz in the drinking water (Figure 4F). Together, these data demonstrate that guanabenz shortens the circadian period and disrupts the circadian rhythms of mouse wheel-running behavior. 

### 2.5. Oral Administration of Guanabenz Disrupts Circadian Oscillations of Clock Protein Per1 and Per2 in the SCN

**Figure 5:**
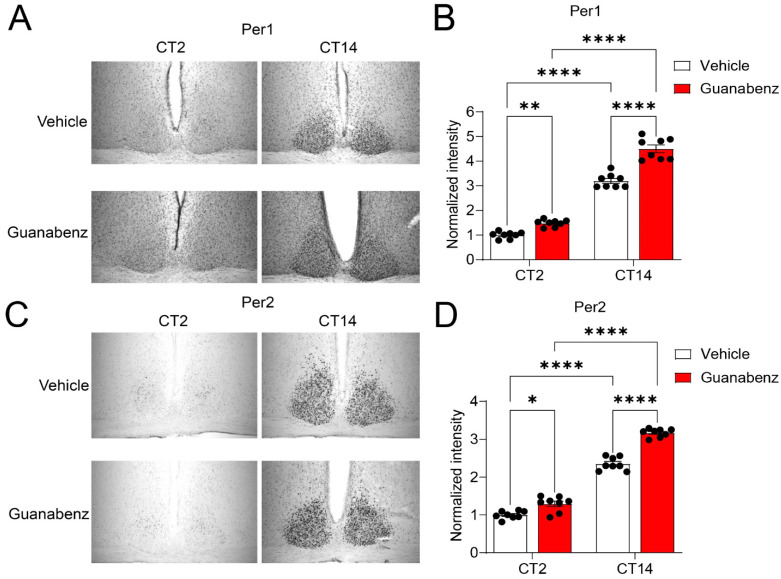
Guanabenz treatment increases Per1 and Per2 levels in the SCN: (**A**) Representative microscopic images of immunostaining for Per1 in the SCN. (**B**) A bar graph indicating the quantified staining intensities for Per1. The levels of staining intensity were normalized to the levels of the vehicle treatment group at CT2. Note that guanabenz treatment significantly increased the Per1 expression at both CT2 and CT14 (F_(1,7)_ = 17.39, *p* = 0.0042, two-way ANOVA; CT2: Vehicle vs. Guanabenz, t = 3.503, *p* = 0.0100; CT14: Vehicle vs. Guanabenz, t = 9.401, *p* < 0.0001; Vehicle: CT2 vs. CT14, t = 15.88, *p* < 0.0001; Guanabenz: CT2 vs. CT14, t = 21.78, *p* < 0.0001; Fisher’s LSD post hoc test). (**C**) Representative microscopic images of immunostaining for Per2 in the SCN. (**D**) A bar graph indicating the quantified staining intensities for Per2. The levels of staining intensity were normalized to the levels of the vehicle treatment group at CT2. Note that guanabenz treatment significantly increased the expression level of Per2 at both CT2 and CT14 (F_(1,7)_ = 15.50, *p* = 0.0056, two-way ANOVA; CT2: Vehicle vs. Guanabenz, t = 3.110, *p* = 0.0171; CT14: Vehicle vs. Guanabenz, t = 8.679, *p* < 0.0001; Vehicle: CT2 vs. CT14, t = 14.35, *p* < 0.0001; Guanabenz: CT2 vs. CT14, t = 19.92, *p* < 0.0001; Fisher’s LSD post hoc test). In (**B**) and (**D**), data are shown as individual values and mean ± SEM. * *p* < 0.05, ** *p* < 0.01, **** *p* < 0.0001.

To investigate the mechanisms underlying the effects of circadian disruption by guanabenz, we examined circadian oscillations of the clock protein Per1 and Per2 in the SCN as indicators of the molecular clock function. Using immunostaining, we found robust oscillations of Per1 and Per2, indicated by low levels at CT (circadian time) 2 and high levels at CT14 in the control mice. Interestingly, we found that Per1 and Per2 were moderately but significantly increased at both CT2 and CT14 in the mice treated with guanabenz as compared to the vehicle-treated mice (Figure 5). Notably, the cellular expression patterns of Per1 and Per2 at CT2 were also changed through guanabenz treatment. Per1- or Per2-positive cells were more diffusively distributed in the SCN of guanabenz-treated mice, as compared to the clustered patterns in the SCN of vehicle-treated mice (Figure 5A,C), indicating circadian disruption by guanabenz at the cellular level. Together, these results indicate that circadian oscillations of clock proteins are disrupted by guanabenz, which may underlie the disruption in circadian rhythms of behavior in guanabenz-treated mice.

## 3. Discussion

Circadian clock dysfunction has been linked to adverse health outcomes in numerous rodent and human studies. Understanding the role played by the fundamental cellular signaling pathways in the clock is critical for developing new therapeutic strategies to promote body clock function and treat circadian dysfunctions. In the current study, we assessed the effects of a commonly used antihypertensive guanabenz on cellular and behavioral circadian rhythms using a combination of molecular, cellular, and behavioral approaches. We found that guanabenz can exacerbate the ISR by increasing eIF2α phosphorylation in cultured fibroblasts and in mice. The hyperphosphorylation of eIF2α is associated with the shortened circadian period in cells and animals and the disruption of behavioral circadian rhythmicity in mice. At the cellular level, guanabenz administration disrupted circadian oscillations of the clock protein Per1 and Per2 in the SCN master clock. These results uncover, for the first time, the significant effects of guanabenz on the circadian system and indicate that exacerbated ISR disrupts the brain’s clock function.

Recent work has started to uncover the critical role of the ISR in regulating circadian clock physiology. We found that the ISR regulator GCN2 rhythmically phosphorylates eIF2α in the SCN [16]. *Gcn2* deletion leads to a lengthened circadian period and disrupted behavioral rhythms in mice. The level of eIF2α phosphorylation bidirectionally regulates the circadian period in cells and mice. In addition, the phosphorylation of eIF2α promotes mRNA translation of Atf4, which in turn promotes *Per2* transcription [16]. In the fungus *Neurospora crassa*, Karki et al. reported that CPC-3 (the homolog of mammalian GCN2) controls rhythmic protein production and is important for circadian clock function [17]. The circadian clock regulates the rhythmic translation of specific mRNAs through rhythmic eIF2α activity. The same group further found that clock-controlled phosphatase, PPP-1, dephosphorylates and activates eIF2α, leading to increased protein synthesis at night in *Neurospora* [21]. The GCN2 signaling pathway maintains the robust circadian clock function in response to amino acid starvation by controlling histone H3 acetylation at the *frq* promoter [22]. Together, these findings in different organisms indicate that the ISR functions as a physiological mechanism for the cellular clock to integrate its function with cellular stress response.

The ISR level is delicately regulated by a feedback mechanism and is frequently dysregulated in cognitive and neurodegenerative disorders, including Alzheimer’s disease, Parkinson’s disease, amyotrophic lateral sclerosis, etc. [12]. The ISR activates PERK, which phosphorylates eIF2α and regulates mRNA translation. Notably, eIF2α phosphorylation represses general translation but activates mRNA translation of *Atf4*, which in turn activates the transcription of CHOP (C/EBP homologous protein) and GADD34 (growth arrest and DNA damage-inducible protein) [23]. GADD34 directs protein phosphatase 1 (PP1) to dephosphorylate eIF2α, thus forming a negative feedback loop to limit the extent of ISR. Recent studies demonstrated that guanabenz inhibits the GADD34 binding to PP1 and the dephosphorylation of phospho-eIF2α, thereby enhancing the ISR [19]. Aberrant ISR is found in neurodegenerative disorders. For example, elevated phosphorylation of eIF2α is found in the brains of Alzheimer’s disease patients and mouse models of Alzheimer’s disease [24]. Many of these diseases are associated with disrupted daily activity rhythms in patients due to unknown mechanisms [25,26]. The role of pathological ISR in circadian regulation has not been studied. In the current study, we found that high doses of guanabenz can lead to exacerbated ISR and disrupted circadian rhythms in mice, indicating that aberrant levels of ISR can be involved in circadian dysfunction in brain diseases with circadian and sleep disruptions. Notably, α2-adrenoceptor subtype 2A mRNA is found to be expressed in the hypothalamus [27]. Although there is no evidence supporting a significant role for α2-adrenergic signaling in regulating circadian rhythms, our current study cannot rule out the possibility that guanabenz may disrupt circadian rhythms through its binding to α2-adrenoceptors.

In summary, our study uncovers unexpected detrimental effects of the antihypertensive guanabenz on the circadian system, which are associated with exacerbated ISR induced by guanabenz administration. Understanding the pathological interactions between ISR and the circadian clock will provide insights into mechanisms of circadian dysfunctions in human diseases in which the ISR is dysregulated. 

## 4. Materials and Methods

### 4.1. Animals and Circadian Wheel-Running Behavioral Assay

Adult (6–8 weeks old) male C57BL/6J mice were individually housed in cages equipped with running wheels, and locomotor activities were recorded as previously described [16]. Wheel rotation was recorded using the ClockLab program (Actimetrics). Mice were first entrained to a standard 12 h/12 h light–dark cycle for 7 d and then released to constant darkness (DD) for 14 d. Regular drinking water was provided. Next, mice were re-entrained to a standard 12 h/12 h light–dark cycle for 7 d, followed by release to DD for 14 d. During this period, guanabenz acetate (Bristol Mayer Squib, Princeton, NJ, USA) was dissolved in distilled water (35 mg/L) and delivered to mice in drinking water. Each mouse received guanabenz acetate of approximately 8 mg/kg/d. The dose was determined based on a previous study indicating that 4–16 mg/kg treatment with guanabenz in mice would likely achieve CNS levels sufficient to modulate the ISR [28]. Wheel-running activities were analyzed using the ClockLab Analysis program (Actimetrics, Wilmette, IL, USA). All experimental procedures were approved by the Institutional Animal Care and Use Committee at the University of Minnesota (1910-37464A, 11 October 2022). 

### 4.2. Brain Tissue Processing

Mice were euthanized via cervical dislocation and decapitation. Brain tissue was promptly harvested and prepared for either immunostaining or Western blotting using the methods previously described [28]. Briefly, to prepare for immunostaining, the brains were sliced into 1 mm sections using the acrylic mouse brain slicer (Zivic instruments, Pittsburgh, PA, USA) and fixed in 4% paraformaldehyde for a duration of 6 h at room temperature. After fixation, the brain slices were moved to a solution of 30% sucrose for dehydration at 4 °C overnight. Subsequently, the slices were cut into 40 µm sections using a Leica SM2010R sliding microtome. To prepare for Western blotting, brain tissue was rapidly frozen in dry ice and stored at −80 °C until the proteins were extracted.

### 4.3. Cell Culture

The 3T3 fibroblasts expressing a *Per2-dLuc* reporter [29] were cultured in the Dulbecco’s modified Eagle’s medium (DMEM) supplemented with 10% FBS and 1X penicillin–streptomycin at 37 °C under a humidified 5% CO_2_ atmosphere. For guanabenz treatments, the cells were plated on 100 mm culture dishes at a density of 3 × 10^6^ cells/dish. On the third day, or when the cell confluence reached 80%~90%, the medium was replaced with a new medium containing various concentrations of guanabenz. After 8 h of treatment with guanabenz, the cells were harvested and stored at −80 °C for protein extraction as reported [29].

### 4.4. Western Blotting

For protein extraction, brain tissue was homogenized using a pestle grinder (Fisher Scientific Limited, Nepean, ON, Canada), followed by treatment with a lysis buffer. Western blotting was carried out as reported [29]. Briefly, protein extracts were loaded onto a 10% SDS-PAGE gel for electrophoresis, and the protein blots were transferred onto polyvinylidene difluoride membranes (Immobilon-P, Merck Millipore Ltd., Carrigtwohill, Ireland). After blocking with 10% skim milk (Fisher Scientific, Fair Lawn, NJ, USA), the membranes were incubated at 4 °C overnight in PBST (PBS with 1% Triton X-100) containing 5% BSA along with the antibody targeting eIF2α (1:300, SC-133132, Thermo Fisher Scientific); p-eIF2α (Ser51) (1:1000, CST3398S, Cell Signaling Tech, Danvers, MA, USA); S6 (1:1000, SC-74459, Santa Cruz Biotechnology, Inc., Dallas, TX, USA); p-S6 (Ser240/244) (1:1000, CST2215, Cell Signaling Tech); or α-tubulin (1:1000, I1224-1-AP, Proteintech, Inc., St. Louis, IL, USA). Subsequently, the membranes were incubated for 1.5 h in PBST (with 5% skim milk) containing an HRP-conjugated secondary antibody (1:5000, donkey anti-rabbit: NA934V; sheep anti-mouse: NA931V, GE Healthcare, Piscataway, NJ, USA). After each antibody treatment, the membranes underwent a minimum of three wash cycles (10 min/wash) using PBST. Chemiluminescence was developed using Western Lightning Chemiluminescence Reagents (PerkinElmer, Inc., Waltham, MA, USA) and captured using X-ray films. The developed films were scanned into digital images, and blot densities were quantified using Adobe Photoshop software (Adobe Systems Incorporated, San Jose, CA, USA).

### 4.5. Immunostaining and Imaging Analysis

For immunohistochemical staining, the sections underwent an initial treatment with PBS containing 0.3% H_2_O_2_ and 20% methanol for 30 min to enhance tissue permeability and deactivate endogenous peroxidases. Following this, the sections were blocked for 1 h using 10% goat serum in PBS and then incubated overnight at 4 °C with rabbit anti-Per1 (1:3000, AB2201, Millipore, Burlington, MA, USA) or anti-Per2 (1:2000, AB2202, Millipore, Burlington, MA, USA) antibody, which was diluted in PBS containing 5% goat serum. Next, the tissue underwent a 1.5 h incubation at room temperature with a biotinylated secondary antibody, which was diluted in PBS with 5% goat serum (1:400; Vector Laboratories, Newark, CA, USA). Following that, the tissue was treated with an avidin–biotin HRP complex (Vector Laboratories, Newark, CA, USA) for 1 h. After each labeling step, the sections were washed in PBS three times, each lasting 10 min. The resulting signal was made visible by employing a nickel-intensified DAB substrate (Vector Laboratories, Newark, CA, USA). Afterward, the sections were mounted on gelatin-coated slides using Permount mounting media (Fisher Scientific, Houston, TX, USA).

Bright-field images were captured at 10X magnification using an inverted DMi8 Leica microscope (Leica, Wetzlar, Germany) with a digital camera. The Per1 and Per2 staining intensities were quantified using Adobe Photoshop software (Adobe Systems Incorporated, San Jose, CA, USA). Group differences were determined using two-way ANOVA. 

### 4.6. Bioluminescence Recording and Data Analysis

For bioluminescence recordings, the 3T3 fibroblasts expressing *Per2-dLuc* reporter cells were plated in 35 mm dishes. Once the cell confluence reached 80~90%, the cells were washed with 1X PBS, and the recording medium (DMEM, 10% FBS, 1X penicillin–streptomycin, 50 mM HEPES pH7.5, 1 mM luciferin) was added. The dishes were then placed in a LumiCycle luminometer (Actimetrics). The 3T3 *Per2-dLuc* cells were treated with guanabenz (5 µM or 10 µM) in the recording medium. Bioluminescence recordings were analyzed using the LumiCycle Analysis program (Actimetrics, Inc.) as previously described [29]. For period length analysis, the raw data were fitted to a linear baseline, and the data subtracted from the baseline were fitted to a sine wave (damped), from which the period length, goodness-of-fit value, and damping constant were determined. All samples showed sustained rhythms and a goodness-of-fit of >80% was achieved. For amplitude analysis, baseline-subtracted data (polynomial order = 1; days 3–6 of recording data) were fitted to a sine wave, from which the amplitude was determined using Sin Fit. One-way ANOVA was used to compare differences among different treatment groups.

### 4.7. Statistical Analysis

Statistical analysis was executed, and graphical representations were generated using GraphPad Prism 7 software (GraphPad Software, La Jolla, CA, USA). Statistical significance was established for *p* < 0.05.

## Data Availability

All data presented in this study are available upon reasonable request from the corresponding author.

## Supplementary Materials

The following supporting information can be downloaded at: https://www.mdpi.com/article/10.3390/clockssleep5040043/s1, Figure S1: Full-length western blotting images.
