# Supplementary figures and images for: The Antihypertensive Guanabenz Exacerbates Integrated Stress Response and Disrupts the Brain Circadian Clock
Source: Clocks Sleep. 2023 Oct 31;5(4):639–50. doi: 10.3390/clockssleep5040043 (PMC10660470; doi:10.3390/clockssleep5040043)

**Fig. 1A**

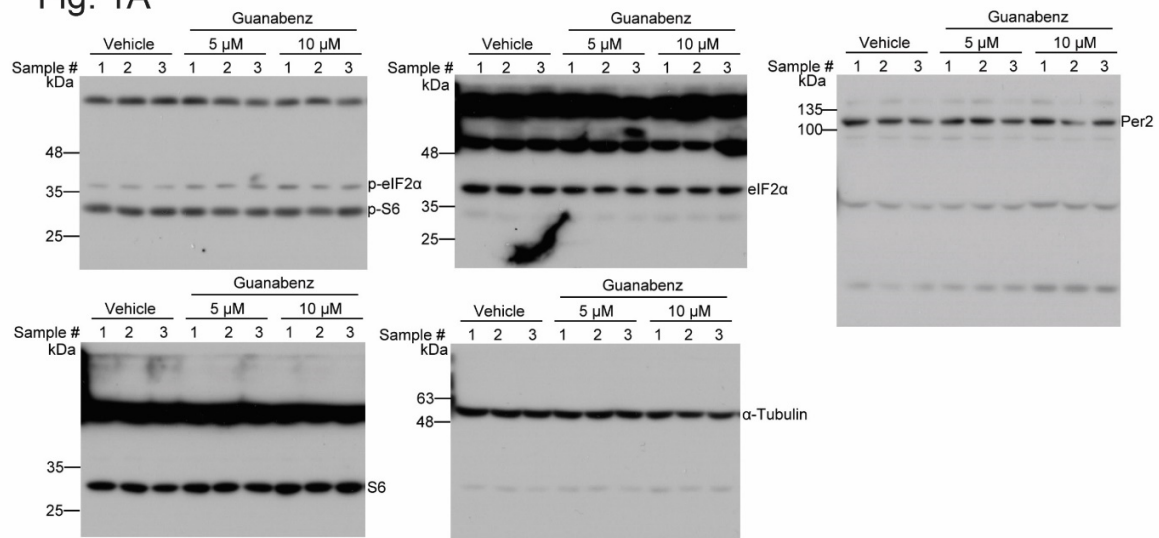

**Fig. 3A**

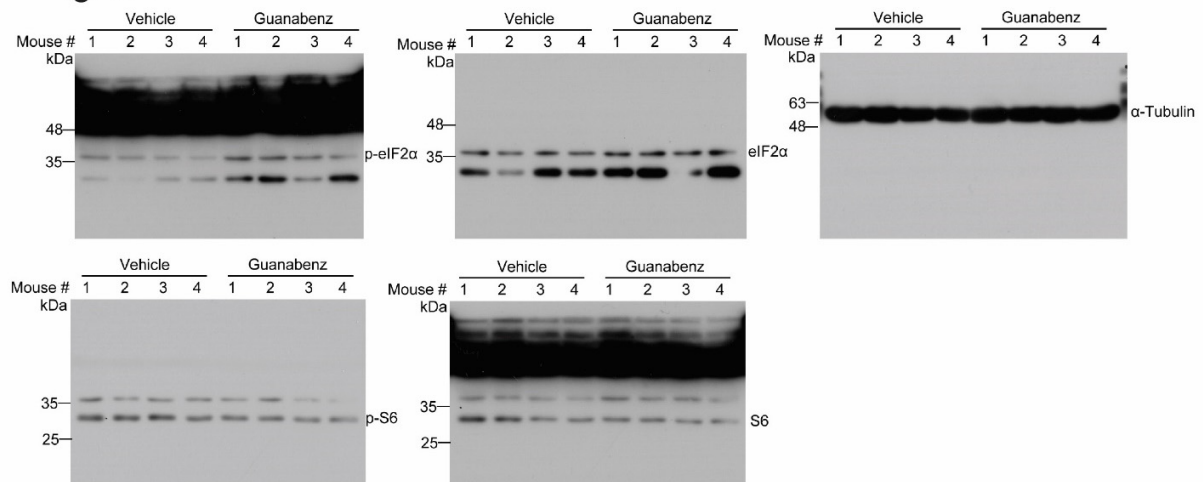

**Supplementary Figure S1. Full-length western blotting images.**

Supplement: Supplementary file 1 [file clockssleep-05-00043-s001.zip › clockssleep-2588879-supplementary.pdf]
